# Investigating the dynamics of surface-immobilized DNA nanomachines

**DOI:** 10.1038/srep29581

**Published:** 2016-07-08

**Authors:** Katherine E. Dunn, Martin A. Trefzer, Steven Johnson, Andy M. Tyrrell

**Affiliations:** 1 Department of Electronics, University of York, Heslington, York, YO10 5DD, UK.

## Abstract

Surface-immobilization of molecules can have a profound influence on their structure, function and dynamics. Toehold-mediated strand displacement is often used in solution to drive synthetic nanomachines made from DNA, but the effects of surface-immobilization on the mechanism and kinetics of this reaction have not yet been fully elucidated. Here we show that the kinetics of strand displacement in surface-immobilized nanomachines are significantly different to those of the solution phase reaction, and we attribute this to the effects of intermolecular interactions within the DNA layer. We demonstrate that the dynamics of strand displacement can be manipulated by changing strand length, concentration and G/C content. By inserting mismatched bases it is also possible to tune the rates of the constituent displacement processes (toehold-binding and branch migration) independently, and information can be encoded in the time-dependence of the overall reaction. Our findings will facilitate the rational design of surface-immobilized dynamic DNA nanomachines, including computing devices and track-based motors.

DNA has been used to construct a wide variety of synthetic structures and devices at the nanoscale, including two- and three-dimensional objects^1^,^2^,^3^,^4^,^5^,^6^,^7^, dynamic machines^8^ and nanorobots^9^, and DNA-based systems can also perform computation^10^,^11^,^12^,^13^. In many DNA devices, dynamic switching is driven by the phenomenon of toehold-mediated strand displacement^14^,^15^,^16^,^17^, in which a double-stranded DNA molecule with a short single-stranded region is disassembled as a result of hybridization with an invading DNA strand, forming a more energetically favourable product. There is now increasing interest in surface-based assembly of DNA objects^18^ and the operation of dynamic DNA machines on surfaces^19^, including DNA walkers that travel along immobilized tracks^20^,^21^,^22^,^23^. In general, surface-immobilization enables spatial localization of molecular components, which has particular benefits for DNA computing, because it enables higher speed, sharper switching and greater modularity, while offering improved potential for parallel processing over ‘global’ solution-phase approaches^24^. A range of computational machines designed to operate on surfaces have been constructed using DNA, including logic gates^25^ and other information processing elements^26^,^27^,^28^. Muscat *et al*. presented designs for a range of localized circuits based on cascades of DNA hairpins attached to a DNA tile, where the hairpins could be opened by input or fuel strands^29^. Fluorescence measurements of strand displacement cascades on DNA origami tiles revealed that the performance was optimal when the sender and receiver gates were separated by a distance short enough to permit direct physical contact between the gates, but long enough to prevent displacement from occurring in the absence of an input^30^. It has also been shown theoretically that DNA walkers^22^ can be used to construct circuits capable of evaluating any Boolean function^31^.

However, molecular crowding can play an important role in the behaviour of surface-immobilized molecules, which are typically more densely packed than their solution-phase counterparts. For instance, it has been shown that the conditions required to induce a structural transition in a synthetic peptide are altered by molecular crowding when the peptides are confined within a surface-immobilized monolayer^32^. DNA hybridization is also known to proceed more slowly on surfaces than in solution^33^ and the response of electrochemical DNA sensors has been found to depend strongly on the surface density^34^. The operation of restriction enzymes diffusing in two dimensions within a DNA monolayer can be inhibited if the density of DNA molecules is too high^35^, and it has also been established that a surface-immobilized DNA stem-loop structure can be either stabilized or destabilized as a result of crowding, depending on the conformation of the surrounding molecules^36^.

Despite these findings and the interest in surface-phase dynamic DNA machines, the effects of surface-immobilization on the kinetics of dynamic DNA processes such as strand displacement have not yet been fully investigated^37^,^38^,^39^. To address this, we undertook a detailed study of the mechanism and kinetics of strand displacement on-surface using quartz crystal microbalance with dissipation monitoring (QCM-D)^40^, a technique which involves the use of acoustic waves to probe surface-immobilized molecules, providing real-time information on their mass and conformation.

Our molecular machine consists of a 16-base pair double-stranded domain (duplex) with a 16-nucleotide single-stranded overhang (‘toehold’ domain). The blunt end carries a thiol modification for oriented immobilization on gold - a common strategy for the formation of self-assembled monolayers^41^. As illustrated in Fig. 1a, the state of the machine is changed by toehold-mediated strand displacement, which begins when a single-stranded invader binds to an immobilized machine, through hybridization of the invader’s toehold binding domain with the complementary toehold. If the invader’s displacing domain is complementary to the corresponding target domain, branch migration ensues, leading to dissociation of double-stranded waste product, leaving an immobilized single-stranded molecule. By revealing how the dynamics of displacement depend on toehold length, toehold G/C content, invading strand concentration and complementarity of the displacing domain, our results will enable the rational design of DNA machines specifically for surface operation, and facilitate optimization of the surface chemistry.

## Results

### Using QCM-D to study surface-immobilized DNA machines

**Figure 1:**
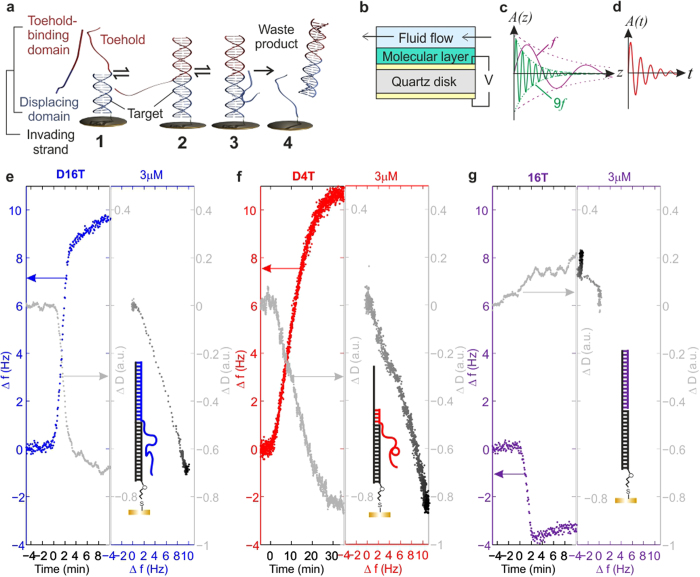
Studying a surface-immobilized DNA nanomachine using a Quartz Crystal Microbalance with Dissipation Monitoring. (**a**) A simple surface-immobilized DNA molecular machine based on toehold-mediated strand displacement. The toehold binding domain of the invading strand binds to the toehold of an immobilized construct (1) to form the displacement complex (2). Branch migration occurs (3), and the invader displaces the incumbent strand (binding to the target domain), leading to release of double-stranded waste product (4). (**b**) Principle of Quartz Crystal Microbalance with Dissipation monitoring (QCM-D), the technique we used to study the nanomachine depicted in part (**a**). A QCM-D sensor consists of a piezoelectric quartz disk with a gold electrode on each side. The application of an AC voltage drives oscillation, and the resulting acoustic wave propagates through a surface-immobilized molecular layer and into solution. (**c**) As indicated, lower frequency waves propagate further into solution (*z* denotes distance from surface); the penetration depth is proportional to 1/√*f*, where *f* denotes the frequency of the wave. (**d**) For each data point, the energy dissipated by the acoustic wave is measured by fitting the exponential decay of the oscillation (shown in this sketch; *t* denotes time) after the drive voltage is switched off, as described in the text. Changes in frequency and dissipation reflect the mass and structure of the immobilized molecular layer, respectively. (**e**–**g**) Strand displacement and toehold-domain binding on a surface, as measured with QCM-D: changes in frequency (Δ*f*) and dissipation (Δ*D*) measured for three different invading strands. (**e**) D16T (blue strand in sketched construct), which has a 16nt toehold binding domain and a 16nt displacing domain. (**f**) D4T (red strand), which has a 4nt toehold binding domain and a 16nt displacing domain. (**g**) 16T (‘toehold-only’ strand, purple), which has a 16nt toehold binding domain and does not have a displacement domain. Dissipation data has been smoothed using a 15-point adjacent averaging filter. In Δ*D*-Δ*f* plots, each point represents a specific structural configuration of the immobilized layer and reveals mechanistic details of the displacement process. Darker points: later time.

We assembled the nanomachines into a monolayer on the surface of a QCM-D sensor, which consists of a gold-coated piezoelectric quartz disk with an electrode on either side (Fig. 1b). To allow controlled delivery of samples to the sensor surface, the sensor was mounted in a flow-cell in which the temperature was maintained at 16 ± 0.02 °C. Here, an AC voltage applied across the gold electrodes forces the piezoelectric sensor to resonate. The acoustic waves generated by the oscillating crystal propagate through the molecular layer immobilized on the sensor surface and into the solution (Fig. 1c). A change in the mass of material deposited on the sensor leads to a shift in the frequency of oscillation, *f*, relative to the original baseline frequency. The change in frequency, Δ*f*, is related to the mass change of the molecular film according to the Sauerbrey equation^42^, Δ*m* = −*C* Δ*f* / *n*, where Δ*m* is the change in the density of immobilized mass (in ng cm^−2^), *n* is the overtone number and *C* is a constant of value 17.7 ng cm^−2^ s^−1^. It is important to note that the Sauerbrey equation assumes a uniform and rigid molecular film.

The sensitivity of the quartz crystal microbalance to immobilized mass has been used to probe hybridization of nucleic acids on surfaces^43^, and more recent studies have demonstrated that QCM-D can also be used to examine the conformation or secondary structure of DNA molecules^44^,^45^, by measuring the energy dissipated by the acoustic wave as it propagates through the layer and into solution^40^. The dissipation, *D*, is defined as *D* = 1*/Q*, where *Q* is the quality factor of the resonator. Here, *Q* = π*f*τ, where τ is the characteristic time over which the amplitude of the acoustic wave decays following removal of the drive voltage (Fig. 1d). Shifts in dissipation and frequency (Δ*D* and Δ*f* respectively) can be measured as a function of time, where Δ*D* reflects changes in the viscoelasticity of the molecular layer and thus can be linked to changes in molecular conformation. For viscoelastic layers where Δ*D* > 2.0 a.u^46^, the Sauerbrey equation is no longer valid (see Supplementary Discussion 2) and Δ*f* and Δ*D* become coupled, but it can be taken as an approximation that Δ*f* mainly reflects changes in surface mass and Δ*D* is related primarily to the viscoelasticity of the surface-immobilized layer. The penetration depth of an acoustic wave is inversely proportional to √*f* (Fig. 1c), which means that recording Δ*f* and Δ*D* at multiple frequencies (overtones) yields additional information about the structure of the immobilized layer at different distances from the sensor surface. For example, if two processes occur concurrently but at different domains within the DNA layer, the Δ*f* and Δ*D* dynamics will be overtone-dependent.

All experiments were based on the same target strand, except when the GC-content of the toeholds was varied (all sequences are given in Supplementary Table 1). The toehold length was varied by truncating the invader. We found that the molecular density of the DNA layer immobilized on the sensor surface via gold-thiol bonds was approximately 10^12^ machines per cm^2^ (calculation given in Supplementary Discussion 2). This is in good agreement with published values^33^ for the density of a double-stranded DNA monolayer of 3 × 10^12^ molecules cm^−2^. At this density, the intermolecular separation is a few nanometres, and the machines are therefore sufficiently close to be affected by intermolecular interactions, being approximately 9 nm long on average (calculation given in Supplementary Discussion 2).

Our QCM-D measurements confirm that the immobilized nanomachine functions effectively as a molecular switch. Specifically, on-surface displacement for invaders with 16nt or 4nt toehold binding domains (Fig. 1e,f) causes an increase in Δ*f*, due to loss of immobilized mass as the waste product dissociates. Simultaneously, Δ*D* falls as the surface-immobilized DNA molecules collapse into a compact conformation due to the reduced persistence length associated with single-stranded DNA^47^. We found that displacement occurs comparatively slowly in these surface-immobilized machines, partly because invaders must diffuse to the surface before the reaction can commence.

### Kinetics of strand displacement on surfaces and the effect of intermolecular interactions

In solution, it is common for the kinetics of DNA displacement to be described using a first order exponential function. However, this function is a poor fit for displacement in the high-density DNA layer of Fig. 1 (see Supplementary Fig. 2). It is therefore inappropriate to use a simple, exponential kinetic model to fit the data for this surface, at this density of molecules. Instead, we observed that the main part of the transition was described very well by a linear function, and consequently we estimated the reaction rate, *v*_*n*_, for each overtone number *n* by performing linear fits to Δ*f(t)* (Supplementary Discussion 2), thus enabling a quantitative comparison of the kinetics between experiments. A full description of our fitting process is provided in the Supplementary Information, including statistical tests that justify our procedure (see Supplementary Discussion 2, Supplementary Figs 3–5 and Supplementary Table 3).

A theoretical description of the underlying physics of strand displacement on surfaces is beyond the scope of this paper. However, it has already been established that interactions between identical immobilized DNA stem-loop structures can lead to broadening of the unfolding transition observed in urea-melt data, due to anti-cooperative effects^36^, and we suggest that related phenomena may be responsible for the linear behaviour of Δ*f(t)* observed in the more dense DNA layers. In the initial state, the molecular machines are sufficiently closely packed that displacement proceeds in the presence of intermolecular interactions. Following strand displacement, the single-stranded molecule that remains on the surface will collapse into a more compact configuration, and this is expected to reduce the probability that it will interact with its neighbours. Consequently, it is possible that conformational switching in one machine facilitates invader binding in the next, and this could be the cause of the change in the shape of the kinetics. This is supported by our experimental studies into the dynamics of strand displacement as a function of the surface density of DNA machines, controlled by co-immobilizing with mercaptohexanol (MCH). As we increased the concentration of MCH, and thus decreased the density of immobilized DNA, we found that the shape of the kinetics began to follow a first order exponential function, typical of displacement in solution (Supplementary Fig. 6). While various models have been developed to describe strand displacement in solution^15^,^16^,^48^, to the best of our knowledge these have not yet been adapted for surface-immobilized systems to account for the significant differences in the local environment of the molecules, including electrostatic interactions and molecular crowding.

### Controlling the rate of strand displacement

**Figure 2:**
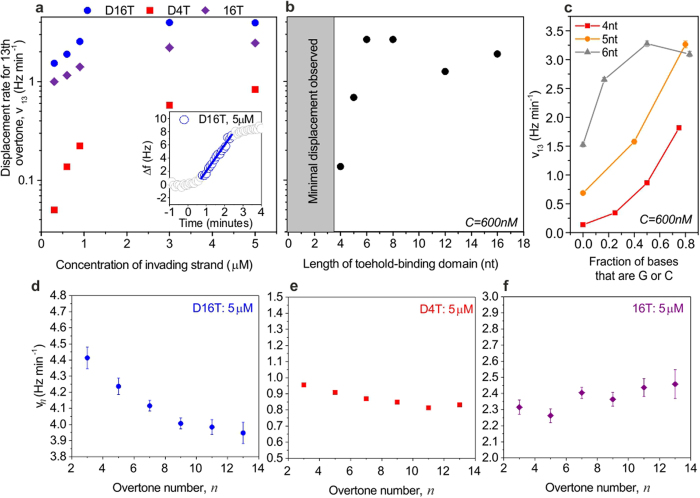
Strand displacement rate, measured for different invader concentrations, toehold lengths, GC content and overtone numbers. (**a**) Rate of strand displacement, *v*_13_, as a function of invader concentration, for the three invader strands introduced in Fig. 1. (**b**) Rate of strand displacement as a function of length of toehold binding domain, where the displacement domain of the invading strand was 16nt long and the invader concentration was 600 nM. The sequence of the invader was obtained by truncating D16T. In both (**a**,**b**), data corresponds to the thirteenth overtone, which has the shortest penetration depth and is therefore least sensitive to the bulk solution. (**c**) Rate of strand displacement, *v*_13_, for invader strands with toehold binding domains of three different lengths, as a function of the fraction of bases in the toehold binding domain that are G or C. Here, a value of 0.2 indicates that 20% of the bases in the domain are G or C, while the remaining 80% are A or T. (**d–f**) Overtone-dependence of displacement or binding rate, for the three different invaders. In all graphs, error bars represent standard error on the rate extracted by fitting. Many of the error bars are too small to see; error bars are shown on all points for which they are visible. For each case, the rate was extracted by performing a linear fit to frequency traces (as indicated in inset, part (**a**), and discussed in the Supplementary Information). The displacing domain was the same for all cases.

In solution, the strand displacement rate can be tuned over orders of magnitude through changes to the length, concentration or sequences of DNA molecules. Our data shows that the same factors can be used to control the kinetics of displacement within the highest density DNA layers. Increasing the invader concentration raises the toehold binding rate (16T, Fig. 2a), and consequently displacement rate (D16T, Fig. 2a), until the immobilized machines are saturated, at 3 μM. In solution, adding a base-pair speeds up displacement 10-fold for toeholds below 6nt in length^15^, with no further enhancement for longer toeholds. On-surface displacement exhibits similar behaviour, but here minimal displacement is observed for toehold-binding domains of length 3nt or less with the original sequence (Supplementary Fig. 8), such that it is not possible to perform a fit to the data for these cases. As shown in Fig. 2b, when the toehold-binding domain is 4nt long, the observed value of *v*_13_ is 0.14 Hz/min (at 600 nM), and when the domain length is increased to 5 and 6nt the rate increases to 0.69 Hz/min and 2.65 Hz/min respectively (same concentration). The displacement rate is thus enhanced by a factor of approximately 4–5 for each additional nucleotide. No further enhancement in the rate of displacement is observed for toehold binding domains above 8nt in length.

We observe that the displacement efficiency, *R*_F_, is independent of concentration and length of toehold binding domain in almost all cases (data presented in Supplementary Fig. 7), suggesting that reactions proceed to completion. Here, the displacement efficiency is defined as *R*_F_ = Δ*f*_displaced_/Δ*f*_immobilized_, where Δ*f*_displaced_ is the frequency shift (in Hz) observed as a result of displacement, and Δ*f*_immobilized_ is the shift observed due to immobilization.

In general, the free energy associated with the formation of a G-C base pair is higher than that for an A-T base pair^49^, due to the number of hydrogen bonds formed. The stability of a DNA duplex of a given length thus increases significantly as the percentage of G/C bases is increased, and in solution this can have a considerable effect on the rate of strand displacement^15^. We have therefore examined the effect of G/C-content on displacement rate in our immobilized nanomachines. For toeholds of 4 and 5nt we observe that the rate is enhanced for invading strands with a high fraction of G/C bases (Fig. 2c). Here, toehold binding becomes more favourable and therefore occurs more rapidly as the number of G/C bases is increased. A similar increase in rate is also observed for toeholds of 6nt but here the rate saturates at approximately 3 Hz min^−1^ (Fig. 2c) for strands with a G/C content ≥50%, because the reaction is fundamentally limited by the rate at which invader strands can associate with immobilized machines. We also find that strand displacement with a 3nt toehold can be successful when the sequence of the toehold is ‘CCC’, with a rate of approximately 1.7 Hz min^−1^ (Supplementary Fig. 9). Changing the composition of a DNA strand therefore provides another way to control the rate of displacement on surfaces.

The process of displacement on surfaces can be examined in more depth using the overtone-dependence of the penetration depth of an acoustic wave, which causes measured parameters to reflect the changes in the mass distribution within the layer that occur during displacement. For instance, the rate, *v*_*n*_, for strand displacement with a 16nt toehold depends on overtone number, *n* (Fig. 2d). Here, toehold binding occurs at the interface between the molecular layer and solution, while dissociation mainly affects the structure near the surface. Lower overtones are sensitive to both processes, yielding faster rates than high overtones that are more sensitive to the final collapse of the immobilized single strand. The overtone-dependence of *v* is minimal for invaders with short toehold binding domains (Fig. 2e), which induce only small conformational changes at the solution-layer interface.

Displacement cannot be initiated by an invader which comprises only a toehold binding domain and does not have a displacing domain. Here, Δ*f* decreases as the invader binds to the toehold and the immobilized mass increases (Fig. 1g). The rate associated with toehold-only binding is overtone-independent (Fig. 2f) because only one process occurs. Although toehold-only binding leads to a shift in frequency, the dissipation for the thirteenth overtone remains approximately constant (Fig. 1g). This high frequency overtone has the shortest penetration depth and is therefore insensitive to the conformation of the toehold binding domain, which is located at the solution-layer interface, comparatively far away from the surface. In contrast, Δ*D* increases for lower acoustic frequency overtones, which propagate further and thus probe restructuring of the toehold domain following hybridization (Supplementary Fig. 10).

### Relative rates of toehold binding and branch migration

Although we observe a decrease in frequency (increase in mass) when the toehold-only strand (16T) binds, (Fig. 1g), there is no evidence of an equivalent initial increase in mass following binding of an invading strand containing both toehold and displacing domains (Fig. 1e,f). This is a surprising result, because an increase in mass would be expected from the formation of an intermediate state in which the toehold of the invader had bound at least partially to the target but branch migration had not yet occurred. The absence of such a mass increase suggests that very few machines dwell in this state for a measurable length of time. In our experiments, frequency and dissipation shifts are recorded every few seconds, and our observation therefore implies that branch migration is much faster than toehold binding.

If branch migration occurred at a comparable rate to toehold binding, we would expect our QCM-D traces to exhibit two separate phases. In the first phase, the frequency would decrease as the surface-immobilized mass increased due to toehold binding, and in the second phase the frequency would increase due to the loss of mass that follows dissociation of waste. These two phases would give rise to two distinct regions in the Δ*D*−Δ*f* plot^50^, as observed in experiments discussed later in the paper, where we reduced the rate of branch migration by introducing mismatched bases. In contrast the Δ*D*−Δ*f* plot for a perfectly complementary invader is approximately linear, characterized by a single gradient over the entire displacement process (Fig. 1e,f). This is consistent with the hypothesis that a single process is rate-limiting.

Further evidence is provided by analysis of the relative rates of toehold-only binding and the full displacement reaction. When we take into account the relative mass of the toehold-only DNA strand and the target strand, we find that the intrinsic rate of displacement is on average approximately 0.8 times the rate of toehold-only binding (calculation in Supplementary Discussion 3). The fact that these rates are similar indicates that toehold binding is the rate-limiting step in strand displacement.

### The effect of mismatches in the displacing domain

**Figure 3:**
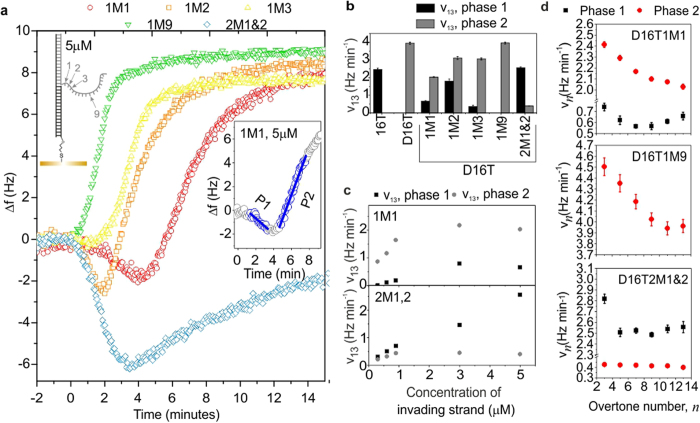
QCM-D results for invading strands with one or more mismatches in the displacing domain. (**a**) Frequency changes as a function of time with mismatch(es) present in the displacing domain of the invading strand at the indicated position(s). Inset–definition of the two phases of displacement (P1 = phase 1 = toehold binding and P2 = phase 2 = dissociation) observed using a mismatched invading strand (shown here for D16T1M1, 5 μM). (**b**,**c**) show the rates extracted by fitting straight lines to Δ*f(t)*, as shown in inset of (**a**), for indicated invading strands. Imperfectly matched invaders are identified by the short name yMx, where y is the number of mismatches, and x gives their location, as indicated in the sketch in part (**a**). All invaders have a 16nt toehold binding domain and the full name (as given in the Supplementary Table 1) is therefore obtained by adding ‘D16T’ to the start of this label. For (**a**) and (**b**), the concentration of invaders was 5 μM. (**a**–**c**) show data for thirteenth overtone. (**d**) Dependence of reaction rate, *v*_*n*_, on overtone number, *n*, for the indicated strands, at a concentration of 5 μM. Where visible, error bars represent the standard error on the fitted rate.

In order to explore the dynamics of displacement on surfaces in more detail, we introduced mismatches in the displacing domain of the invader strand, such that one or two bases in this domain were not complementary to the corresponding bases in the target. In each case, NUPACK^51^ was used to select the mismatch that yielded the duplex with the least favourable free energy of hybridization. The presence of a mismatch introduces an additional energy barrier for branch migration, which must be overcome before branch migration can proceed. This effectively reduces the rate of branch migration without affecting toehold binding. Our QCM-D experiments revealed that the presence of a mismatch at the first and/or second nucleotide of the displacing domain had a strong effect on displacement. Here, the displacement dynamics are non-monotonic (Fig. 3a), comprising two phases. In phase 1, Δ*f* decreases due to the addition of immobilized mass from toehold binding. In phase 2, Δ*f* increases, as mass is lost by dissociation.

Displacement with an invader containing two mismatches is considerably slower than for a fully complementary invader (Fig. 3b,c), because now three base-pairs in the existing duplex must fray^16^ before migration can occur, and many toeholds will be occupied before waste starts to dissociate. The rate of the binding-dominated phase (Fig. 3a, phase 1) is similar to that measured for toehold-only binding (16T, Figs 1g and 3b), and depends strongly on invader concentration, reflecting the concentration-dependent probability of toehold binding. In contrast, the dissociation-dominated phase (Fig. 3a, phase 2), *v*_*13*_ is approximately concentration-independent. Now, branch migration is rate-limiting and is temporally separated from toehold binding. Both the phase 1 and phase 2 reaction rates are essentially independent of overtone number (Fig. 3d), because the two phases are independent, and changes in mass and conformation result from a single process.

The form of the kinetics of displacement for an invader with a double mismatch allows us to estimate the rate of branch migration explicitly. The toehold-binding domain is perfectly matched and binds in the same way as before, and the configuration in which the toehold is bound is regarded as the initial state. For the reaction to proceed, the first two base pairs of the lower duplex must fray, and the mismatched base-pairs must form. This is energetically unfavourable, and may be regarded as a transition state, with an activation energy of ΔG* (measured in J/mol). Subsequently, branch migration and loss of waste occur. According to the Arrhenius equation, the rate of the reaction from the initial state (toehold bound) to the final state (waste dissociated) via the transition state is given by k* = *Ae*^−ΔG*/*RT*^, where *A* is a constant, *R* = 8.31 J mol^−1^ K^−1^ and *T* represents the temperature (in K). The pre-exponential factor may be found by considering the perfectly matched case, in which the activation energy is zero, and here by definition *A* = *k*_*X*_, where *k*_*X*_ is the rate of branch migration and loss of waste. For the double mismatch case the rate k^*^ can be found directly from the data because the toehold binding step is kinetically isolated from the displacement step, and k* is equal to the measured rate of phase 2. From our data (for 5 μM) we obtain a rate of *k*_*13*_ = 0.398 Hz min^−1^, associated with a frequency change of approximately 4.6 Hz, which yields k^*^ = 0.398/4.6 = 0.0866 min^−1^.

The value of ΔG* corresponds to the energy penalty of forming two mismatched bases, relative to the state in which both bases are paired correctly. The hybridization energy is well-known for solution-phase reactions, but the local environment near the surface is likely to have a significant effect on the expected values, and this phenomenon is not yet well-understood. As an estimate, we have adopted the value computed with the online analysis program NUPACK, which uses the solution-phase parameters. This yields a difference in energy between the double-mismatched end product and the perfectly matched end product of 7.69 kcal mol^−1^ = 32 175 J mol^−1^. Substituting the values of ΔG* and k* into the Arrhenius equation (*k*_*X*_ = k*e^ΔG*/*RT*^) allows us to estimate *k*_*X*_, from which we obtain a characteristic time for the process of branch migration and waste loss of approximately 1 ms. This is equivalent to a rate of 16kbp/second, which compares well with literature values^52^.

The displacement rate for an invader with a single mismatch in the first position is also slower than for a fully complementary invader (Fig. 3). Now the rate of phase 2 depends strongly on concentration and overtone (Fig. 3c,d). This resembles displacement for a perfectly matched invader, and implies that branch migration and toehold binding are no longer kinetically isolated, although the displacement dynamics remain non-monotonic, which implies that toehold-binding is not strictly rate-limiting.

The energy barrier introduced by a single mismatch depends strongly on the position of the mismatch in the displacement domain. When the mismatch is half way down the displacing domain (position 9) it has no effect on the kinetics of displacement, and when placed at the third nucleotide it exerts only a weak influence on the dynamics (Fig. 3). This echoes the result of a study performed in solution^53^, which revealed that strand displacement rate can be dramatically affected by the inclusion of a mismatched base, and that the position of the mismatch had a significant effect on the observed rate. However, that work focused mainly on short toehold binding domains, which could dissociate from the toehold before initiation of displacement when there was a mismatch early in the displacing domain. For our study, which used a long toehold binding domain (Supplementary Discussion 1), this may not apply. Detailed theoretical studies would be needed to identify the definitive cause for the connection between the kinetics of on-surface displacement and mismatch position.

**Figure 4:**
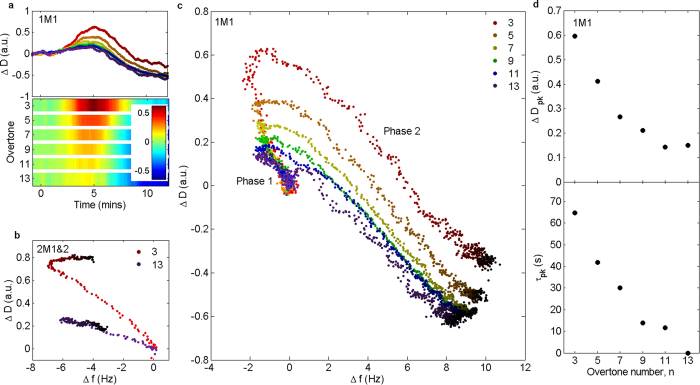
Dissipation changes for invading strands with mismatches in displacing domain. (**a**) Δ*D* as a function of time for all overtones, for D16T1M1 (one mismatch at 1st nucleotide of displacing domain). Upper half – kinetics. Colour code as (**c**). Darker points: later time. Lower half: corresponding surface plot, showing Δ*D* for each overtone. (**b**) Δ*D*-Δ*f* plot for D16T2M1&2 (two mismatches in displacing domain, at 1^st^ and 2^nd^ nucleotides). Darker points: later time. (**c**) Δ*D* as a function of Δ*f* for the different overtones, for D16T1M1. Darker points: later time. (**d**) Value of dissipation peak and time at which it occurs (relative to 13th overtone) as a function of overtone number. In parts (**a**–**c**), Δ*D* was smoothed using a 15-point adjacent averaging filter. All plots: concentration of invading strand was 5 μM.

Mismatched invaders also produce non-monotonic behaviour in *ΔD(t)*, which initially increases as invaders bind, and decreases during dissociation of waste and collapse of immobilized DNA (Fig. 4a). The two phases and the transition between them can be distinguished in Δ*D* −Δ*f* plots (Fig. 4b,c). With two mismatches, the binding and dissociation phases are effectively decoupled and the Δ*D* −Δ*f* plots exhibit two distinct, linear regions separated by a sharp transition (Fig. 4b). For a single mismatch, (Fig. 4c) the transition is more gradual. The turning point in Δ*D* −Δ*f* space shifts with overtone number (Fig. 4a,c) because different overtones are sensitive to different processes (low overtones probe toehold binding and displacement, high overtones are predominantly sensitive to collapse of the DNA following displacement). The time at which Δ*D* peaks is also overtone-dependent, with peak dissipation occurring later for low overtones, once collapse of the immobilized DNA becomes dominant (Fig. 4d).

## Discussion

**Figure 5:**
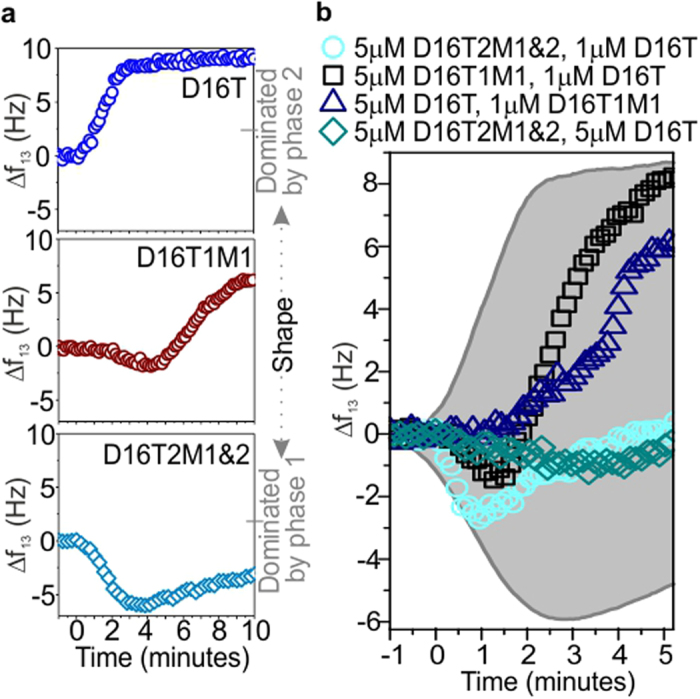
Continuously tuning the shape of the displacement signal. (**a**) Defining the range of displacement shapes. Kinetics measured for indicated strands; shape ranges from phase-1-dominated to phase-2-dominated. (**b**) Measured frequency shift upon addition of indicated mixture of strands. The shaded grey area indicates the range of possible traces. In part (**a**) every 5th point is plotted; in part (**b**) every 3rd point is plotted. All plots show the 13th overtone.

Our results indicate that interactions between surface-immobilized DNA machines affect the kinetics of the strand displacement reaction. Base sequence, length and concentration of invading strands can be used to regulate strand displacement in the nanomachines. Our findings are relevant to the design of DNA motors that move on surfaces and the development of new molecular computing systems. Mismatched bases engineered into the displacing domain of an invader can also change displacement speed and adjust the temporal shape of the signal between the extremes of ‘phase-1-dominated’ and ‘phase-2-dominated’ (Fig. 5a). This could be exploited for detection of single-nucleotide polymorphisms or to develop an alternative approach to DNA computation. The conventional binary formalism defines signals to be ‘on’ or ‘off’, but in a new paradigm, the state of the system could be represented by the functional form of the displacement signal. This would enable multi-valued discrete logic, where the state could be changed by altering a single nucleotide, increasing information encoding efficiency. Alternatively, a continuum of states could be established by tuning the dynamics continuously. In our system this can be achieved simply by controlling the mixture of inputs (Fig. 5b). This result has the potential to underpin phenomenological characterization of unknown mixtures of biomolecules or alternative information processing strategies that overcome the limitations of molecular computation in solution, and will be able to operate beyond the binary logic paradigm.

## Methods

### QCM-D

A Q-sense E4 machine from Biolin Scientific was used, with gold-coated AT-cut quartz sensors, which had a fundamental frequency of 4.95 MHz+/−50 kHz (also from Biolin Scientific, reference number: QSX 301). Sensors were cleaned prior to measurements as follows. Initially they were placed in a UV-ozone cleaner for ten minutes, with the active gold-coated surface facing upwards. The sensors were then successively rinsed in a 2% solution of Hellmanex III and ultrapure MilliQ water (twice), being sonicated for ten minutes in each bath. The sensors were dried with N_2_ gas and subjected to further UV-ozone cleaning (thirty minutes). Next, the sensors were left to soak in 100% ethanol for approximately thirty minutes. Finally they were dried with N_2_ and installed in the flow modules.

Each QCM-D chamber was flushed with ultrapure MilliQ water to enable a stable baseline to be achieved before beginning measurements. All experiments were performed at 16 °C , and this temperature was maintained using the flow module control system. This temperature was chosen to minimize DNA melting when toeholds were short and to ensure that the sample temperature was below ambient, to reduce the risk of bubble formation. The pump flow rate was maintained at a constant value of 20 μL/min.

### Materials

Oligonucleotides were purchased from Integrated DNA Technologies (IDT), all with standard desalting purification except for the thiol-modified strand, for which HPLC purification was used. DNA was resuspended in 1xTE, usually to a concentration of 100 μM (computed using the manufacturer’s quantitation) and stored frozen at −20 °C. Sequences are provided in Supplementary Table 1.

### Typical experiment

A typical experiment was performed as follows. Sensors were cleaned and installed in the flow modules as described above, where four sensors could be used in parallel. After the measurement chamber had been flushed with ultrapure MilliQ water, the experimental buffer (1xTE, 1 M NaCl) was introduced. A baseline was established, before the introduction of a sample containing 300 nM pre-hybridized duplexes, with a thiol modification at one end and a single-stranded extension at the other. After arrival of the sample at the sensor surface, a period of 20–30 minutes was typically required for immobilization. The surface was then backfilled using a freshly prepared 1 mM solution of 6-mercapto-1-hexanol (MCH - irritant) in TE with 1 M NaCl. The MCH is needed to block holes in the incomplete DNA monolayer and to inhibit non-specific DNA adsorption. In the final stage of the experiment, the invading strand was supplied at the specified concentration, in the same buffer. Care was taken at all times to ensure that the salt concentration of all samples was identical.

A time-course of a complete experiment is shown in Supplementary Fig. 1. Control experiments confirmed that no change of state was observed after supplying DNA strands that had no sequence complementarity with the immobilized machines or no toehold (Supplementary Fig. 11).

### Data availability

All experimental data will be made freely available online at the following DOI: 10.15124/158bf570-ad38-43b5-93d5-8c33dc683cc2.

## Additional Information

**How to cite this article**: Dunn, K. E. *et al*. Investigating the dynamics of surface-immobilized DNA nanomachines. *Sci. Rep*. **6**, 29581; doi: 10.1038/srep29581 (2016).

## Supplementary Material

Supplementary Information
