# Supplementary material for: Investigating the dynamics of surface-immobilized DNA nanomachines
Source: Sci Rep. 2016 Jul 8;6:29581. doi: 10.1038/srep29581 (PMC4937388; doi:10.1038/srep29581)
Supplement: Supplementary Information [file srep29581-s1.pdf]

Supplementary Information: Investigating the dynamics of surface-immobilized DNA nanomachines

Katherine E. Dunn, Martin A. Trefzer, Steven Johnson, Andy M. Tyrrell*

Department of Electronics, University of York, Heslington, York, YO10 5DD

*e-mail: katherine.dunn@york.ac.uk

CONTENTS

**Supplementary Table 1**	**DNA sequences**
**Supplementary Table 2**	**Table showing identity of strands used for experiment in which the GC content was varied**
**Supplementary Discussion 1**	**Length of toehold binding domains**
**Supplementary Figure 1**	**Time-course of complete experiment**
**Supplementary Discussion 2**	**Analysis of QCM-D data**
**Supplementary Figure 2**	**Failure of monoexponential fit to describe $\Delta f(t)$**
**Supplementary Figure 3**	**Data analysis- examples of successful fits to $\Delta f(t)$ using linear function**
**Supplementary Table 3**	**Results of fits shown in Supplementary Figure 3**
**Supplementary Figure 4**	**Histogram of residuals for fit to data for D16T (600nM)**
**Supplementary Figure 5**	**Residuals for fit (D16T, 600nM), as function of independent variable**
**Supplementary Figure 6**	**Effect of changing the density of molecules on the surface**
**Supplementary Figure 7**	**Strand displacement efficiency for invaders with no mismatches**

**Supplementary Figure 8**	**Minimal displacement observed when toehold binding domain is shorter than 4nt (with the original target sequence)**
**Supplementary Figure 9**	**Strand displacement observed for invader with a 3nt toehold binding domain that is 100% C**
**Supplementary Discussion 3**	**Calculation of relative rates of strand displacement and toehold binding**
**Supplementary Figure 10**	**Overtone-dependence of dissipation shift observed for binding of toehold-only invading strand**
**Supplementary Figure 11**	**Control experiments**

Supplementary Table 1: DNA sequences.

All sequences are written 5' to 3'. The thiol modifier for the capture strand was supplied in disulphide form, with a three-carbon alkyl chain on either side of the disulphide bond. Naming conventions: if the strand has a displacing domain, the name begins with D. The length of the toehold binding domain is given by the number which follows the D and precedes the T. If there are any mismatches in the displacing domain, these are specified next, in the form [number of mismatches]M[position of mismatches]. Hence, D16T2M1&2 indicates a strand with a displacing domain, a 16 nucleotide long toehold binding domain, and two mismatches in the displacing domain at positions 1 and 2.

SEQUENCE	NAME	DESCRIPTION
ACACGCATACACCCAT-(thiol)	CS	Thiolated capture strand
ATGGGTGTATGCGTGTTTAAAGACCCTAAGCT	16ntOH-RC-CS	Reverse complement of CS, with 16nt overhang
AGCTTAGGGTCTTTAAACACGCATACACCCAT	D16T	Displacing strand with 16nt toehold (reverse complement of 16ntOH-RC-CS)
TAGGGTCTTTAAACACGCATACACCCAT	D12T	Displacing strand with 12nt toehold
GTCTTTAAACACGCATACACCCAT	D8T	Displacing strand with 8nt toehold
CTTTAAACACGCATACACCCAT	D6T	Displacing strand with 6nt toehold
TTTAAACACGCATACACCCAT	D5T	Displacing strand with 5nt toehold
TTAAACACGCATACACCCAT	D4T	Displacing strand with 4nt toehold
TAAACACGCATACACCCAT	D3T	Displacing strand with 3nt toehold
AAACACGCATACACCCAT	D2T	Displacing strand with 2nt toehold
AACACGCATACACCCAT	D1T	Displacing strand with 1nt toehold
ACACGCATACACCCAT	D0T	Displacing strand with 0nt toehold (reverse

		complement of CS)
AGCTTAGGGTCTTTAACCACGCATACACCCAT	D16T1M1	As D16T, but with 1 mismatch at first nt in displacing domain
AGCTTAGGGTCTTTAAAAACGCATACACCCAT	D16T1M2	As D16T, but with 1 mismatch at second nt in displacing domain
AGCTTAGGGTCTTTAAACCCGCATACACCCAT	D16T1M3	As D16T, but with 1 mismatch at third nt in displacing domain
AGCTTAGGGTCTTTAAACACGCATCCACCCAT	D16T1M9	As D16T, but with 1 mismatch at ninth nt in displacing domain
AGCTTAGGGTCTTTAACTACGCATACACCCAT	D16T2M1&2	As D16T, but with 2 mismatches at first and second nts in displacing domain
TTAACCACGCATACACCCAT	4DT1M1	As D4T, but with 1 mismatch at first nt in displacing domain
AGCTTAGGGTCTTTAA	16T	Same toehold as D16T, but no displacing domain
GTCATTTCTCTAAGTA	NC	Completely non-complementary strand
ATGGGTGTATGCGTGTAAAAAAAAAAAAAAAA	16ntOH-RC-CS_ATrich	Strands used for testing effect of varying GC-content
CCCAACACGCATACACCCAT	D4T_GC3	
ATGGGTGTATGCGTGTTGGGTAATAATAATAA	16ntOH-RC-CS_gc3	
CCAAACACGCATACACCCAT	D4T_GC2	
ATGGGTGTATGCGTGTTTGGTAATAATAATAA	16ntOH-RC-CS_gc2	
CAAAACACGCATACACCCAT	D4T_GC1	
ATGGGTGTATGCGTGTTTTGTAATAATAATAA	16ntOH-RC-CS_gc1	
CTCCCACACGCATACACCCAT	D5Tstrong	
ACCAAACACGCATACACCCAT	D5Tint	
CCTCCCACACGCATACACCCAT	D6Tstrong	
TACCCAACACGCATACACCCAT	D6Tint	
TTTTTTACACGCATACACCCAT	D6Tweak	
CCCACACGCATACACCCAT	D3Tstrong	
ATGGGTGTATGCGTGTGGGAGGTAATAATAAT	targetD6Tstrong	
ATGGGTGTATGCGTGTGGGTTAATAATAATAA	TargetD3Tstrong	

DNA sequences are displayed in monospaced font (all characters are the same width) to facilitate comparisons. Sequences were designed manually, and the online nucleic acids package NUPACK was used to evaluate the sequence of domains to reduce or eliminate secondary structure.

Supplementary Table 2: Table showing identity of strands used for experiment in which the GC content was varied

For the experiments in which the GC-content of the toehold was varied, different target strands were used. The table below shows the identity of strands used for each experiment. If an invader strand is not listed below, the target was 16ntOH-RC-CS.

INVADER	TARGET
D5Tstrong	targetD6Tstrong
D5Tint	16ntOH-RC-CS_gc2
D6Tstrong	targetD6Tstrong
D6Tint	16ntOH-RC-CS_gc3
D6Tweak	16ntOH-RC-CS_ATrich
D3Tstrong	TargetD3Tstrong
D4T_GC3	16ntOH-RC-CS_gc3
D4T_GC2	16ntOH-RC-CS_gc2
D4T_GC1	16ntOH-RC-CS_gc1

Supplementary Discussion 1: Length of toehold binding domains

For experiments conducted in solution, it is normal to use toeholds no longer than 7 nucleotides. In our investigation, we used much longer toehold binding domains, of lengths up to 16 nucleotides. When a 16-nucleotide domain hybridizes with its complement, the duplex is highly stable at the measurement temperature (16°C), and this enabled us to perform experiments in which we monitored toehold-only binding, using a strand with no displacing domain. In this way we were able to extract rates for toehold-only binding. Furthermore, the frequency change measured with QCM-D is larger when the mass of the deposited molecules is greater, which enhanced the non-monotonic nature of the data traces measured in the experiments performed using an invader containing a mismatched base in the displacing domain.

Supplementary Figure 1: Time-course of a complete experiment

Frequency and dissipation changes measured for the thirteenth overtone over a representative experiment, showing immobilization, backfilling and displacement using fully complementary invading strand (D16T). The origin was set by the data acquisition software and no post-processing (e.g. smoothing) was performed. Initially the baseline was established using a buffer-only sample consisting only of 1xTE with 1M NaCl. In the second phase, the double-stranded DNA construct under test was immobilised. This was followed by backfilling with 6-mercapto-1-hexanol (MCH) and finally by application of the displacing strand.

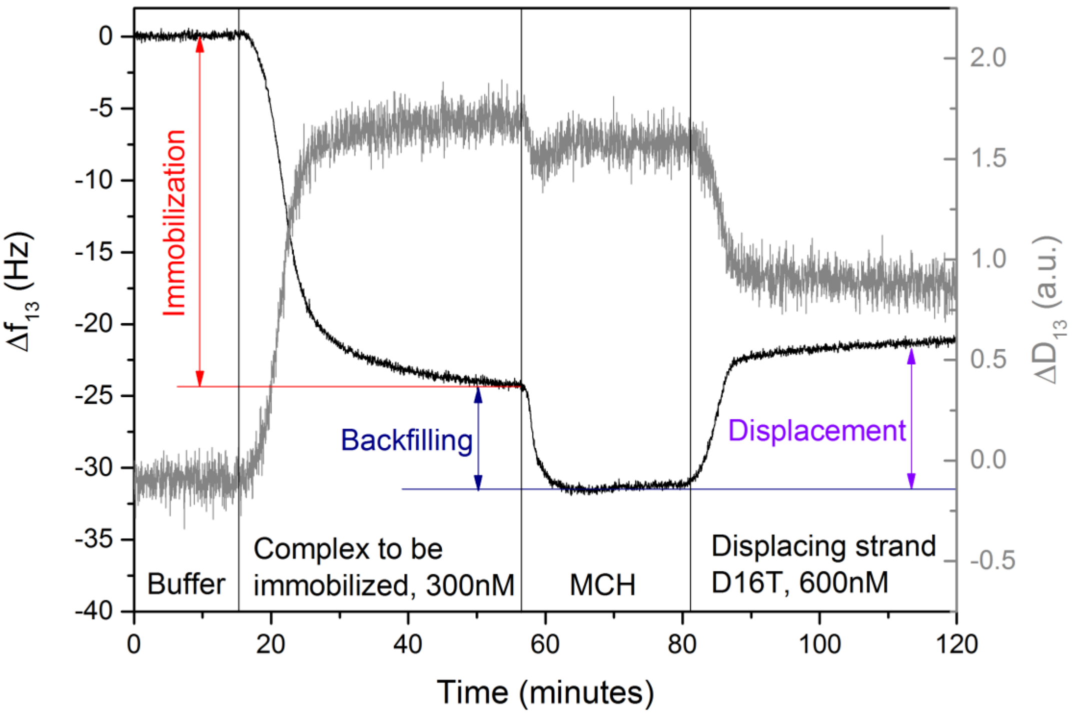

Fig. S 1

Supplementary Discussion 2: Analysis of QCM-D data

If the molecular layer is thin, homogeneous and rigidly attached to the crystal, the Sauerbrey equation holds and:

$$\Delta m = -C\, \Delta f / n \ ,$$

where Δm is the change in the density of immobilized mass (in ng cm^{-2}), Δf is the frequency change, n is the overtone number and C is a constant of value 17.7 ng cm^{-2} s^{-1}. In our experiments, the frequency change following immobilization is approximately 25 Hz (measured with the 13th overtone). According to the Sauerbrey equation, this corresponds to a surface density of 34 ng cm^{-2}. The mass of an immobilized construct is approximately 2.6x10^{-11} ng molecule^{-1}, which suggests that there are of order 1x10^{12} molecules per square cm, with an intermolecular separation of a few nm. For comparison, the length of each machine is approximately 9nm, calculated as follows. Each one consists of a 16bp double-stranded domain and a 16nt single-stranded domain. The double-stranded domain is of length 16x0.34=5.44nm, and the root-mean-square separation of the ends of the single-stranded domain can be estimated to be approximately L_s= 3.5nm by using a freely-jointed chain model. Here, $L_s = b\sqrt{N}$, where b is the Kuhn length of 1.5nm and N is the number of segments, with each segment containing 3nt.

If the helices were immobilized in a hexagonal-close-packed arrangement, such that they were touching each other and no water molecules were trapped between them, the surface density would be approximately 3x10^{13} molecules cm^{-2}. This is calculated as follows. The separation of adjacent helices in this scenario would be 2nm (the helix diameter). The primitive unit cell of the lattice would therefore be composed of two equilateral triangles of side length 2nm, the area of

each being √3 nm^2. Consequently the area per helix would be 2√3 nm^2, and there would be 1/(2√3) helices per nm^2, i.e. 0.29 molecules nm^{-2}.This is an upper limit; in practice the DNA constructs would repel each other due to electrostatic interactions and would be surrounded by a layer of water, which means that they would be more widely spaced than assumed in this calculation. The surface density calculated from the Sauerbrey equation agrees very well with published values for the density of a double stranded DNA monolayer, as discussed in the main paper, but it is important to note that this equation is not very accurate for a viscoelastic monolayer of this type, and the value obtained does in fact depend on the overtone used. For n=3, the surface density is calculated to be 7×10^{12} molecules cm^{-2}, differing by a factor of 7 from the value computed for n=13.

It may be assumed that the Sauerbrey equation breaks down when the dissipation shift exceeds a value of around 2×10^{-6} , as mentioned in the main paper (measurements are usually quoted in arbitrary units, where the factor of 10^{-6} is omitted). This limit is approached or surpassed in the experiments described here, and hence there is not a perfectly linear relationship between the frequency changes measured and the mass immobilized on the sensor, which explains why there are discrepancies in the mass calculations for different overtones.

Inspection of Supplementary Figure 2 reveals that a function containing a single exponential term does not accurately describe the frequency changes observed by QCM-D during strand displacement for a monolayer formed by immobilization of DNA machines followed by backfilling with mercaptohexanol. The fit does not match the data well, and the error on one of

the fit parameters exceeds 200%. In this example, the characteristic time of 1.4 minutes corresponds to a rate constant of k=2.4x10^3 M^{-1}s^{-1} and although the fit is poor, this can be taken to indicate the order of magnitude of the rate of the underlying reaction. The data shown corresponds to one of the fastest reactions measured, but the rate constant k is still three orders of magnitude lower than the corresponding rate in solution.

Attempts to fit a function consisting of the sum of two exponential terms failed, but we observed that it was possible to fit a linear function directly to $\Delta f(t)$. The region of the curve in which Δf depends linearly on elapsed time corresponds to the period during which most of the strand displacement reaction occurs, and the gradient of the straight line (in Hz min^{-1}) is therefore a quantitative measure of the rate of the process.

Selection of datapoints for fitting

The linear region of the trace was identified manually, by visual inspection. The approximate time at which the transition starts is straightforward to find because it corresponds to the arrival of the sample at the sensor, which happens at a known stage of the experiment. The linear regions were then readily identifiable. The quality of the fit was assessed, and the straight line was generally found to pass very close to almost all points, indicating that the fit had worked and appropriate datapoints had been selected. Subsequently, statistical measures were used to confirm this.

We found that our procedure was effective in all cases examined, and representative fits are shown in Supplementary Figure 3, from which it will be seen that the fits match the data closely. Supplementary Table 3 presents the results of the fits, together with measures of the quality of the process. This data reveals that the standard error for the extracted values of v_{13} is usually very small, which demonstrates that the fit is a good description of the data, and an appropriate region of the trace has been chosen. The correlation of the dependent and independent variables can be quantified using the Pearson's correlation coefficient, which is defined for two datasets X and Y, both containing N points, as $r=(\Sigma_i^N (X_i - \langle X \rangle)(Y_i - \langle Y \rangle))/(N\sigma_X\sigma_Y)$, where σ_X and σ_Y represent the standard deviations of X and Y respectively. If the magnitude of r is equal to 1, then X and Y are perfectly correlated. Supplementary Table 3 also shows that the magnitude of this coefficient is generally close to 1, which indicates that the dependent and independent variable exhibit near-perfect correlation, confirming the validity of the fitting approach and our data selection method. Further confirmation can be provided by analysis of the residuals of the fit, as provided below for one example dataset.

Analysis of the residuals of the fit for a representative dataset

The ith residual is defined as $r_i = (\textit{observed value of } y)_i - (\textit{predicted value of } y)_i$, where the predicted value is computed from the fit. If the fitted function is suitable, the deviations from the fit will result solely from stochastic effects and the residuals will therefore be distributed normally around 0. A histogram of the residuals for a representative fit (13th overtone, D16T, 600nM) is presented in Supplementary Figure 4, and it will be seen that the distribution is exactly as expected for a good fit. The distribution of residuals can itself be fitted very well with

a Gaussian that is centred on zero, within the error, and the Anderson-Darling test yields a *p*-value of 0.6162, where there were 73 residuals. This test measures the normality of a dataset. The null hypothesis is that the data comes from a normal distribution, and if the *p*-value is small this hypothesis must be rejected, where the threshold for rejection is typically taken to be 0.05. Thus, obtaining an above-threshold *p*-value in this case indicates that the data is consistent with the hypothesis, which states that the residuals are normally distributed.

Supplementary Figure 5 shows the residuals for this fit as a function of time. When a line of the form $y=mx$ is fitted to this data, the value of m is found to be equal to zero (within the error). This result, combined with visual inspection of the graph, confirms that the residuals exhibit no dependence on time, and this reveals that there is no systematic deviation from linearity within the fitted data. Together with the results confirming that the residuals are normally distributed, this supports our argument that a linear fit is appropriate, and that we have selected the correct region over which to apply the fit.

Note: All experimental data will be made freely available online at the following DOI: 10.15124/158bf570-ad38-43b5-93d5-8c33dc683cc2 .

Supplementary Figure 2: Failure of monoexponential function to describe Δ*f(t)*

Frequency change for strand displacement with D16T invading strand, at a concentration of 5μM. Black circles represent datapoints included in the fit, while grey points were omitted. Red line indicates result of attempt to fit monoexponential function. Fit parameters obtained are given in the plot (left column of numbers: fit parameters, right column: associated errors).

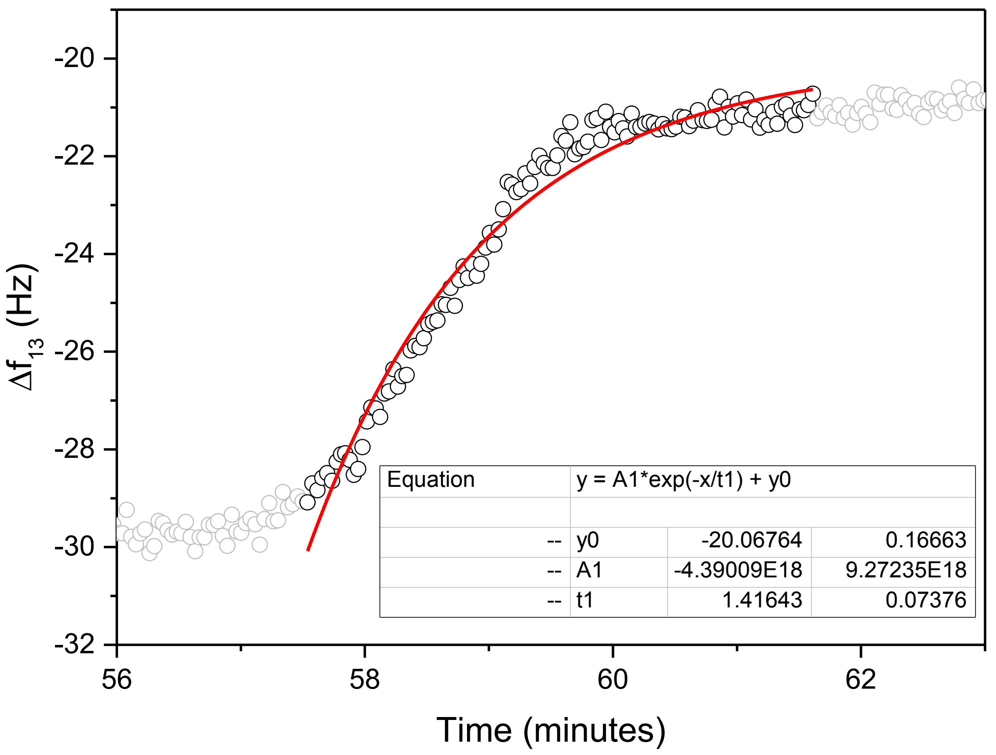

Fig. S3

Supplementary Figure 3: Data analysis – examples of successful fits to $\Delta f(t)$ using linear function

The invading strand used in each case is indicated by the label (where names are as given in Supplementary Table 1). Points in grey were excluded from the fit. For the D4T and D16T2M1&2 figures, some points are omitted from the plot for display purposes but were included in the fit. For the former, every 10th point is shown, for the latter every 5th point is shown. In all graphs, data is presented for all overtones as indicated in the legends. The origin was set by the data acquisition program such that (0,0) corresponded to the start of the experiment before immobilization.

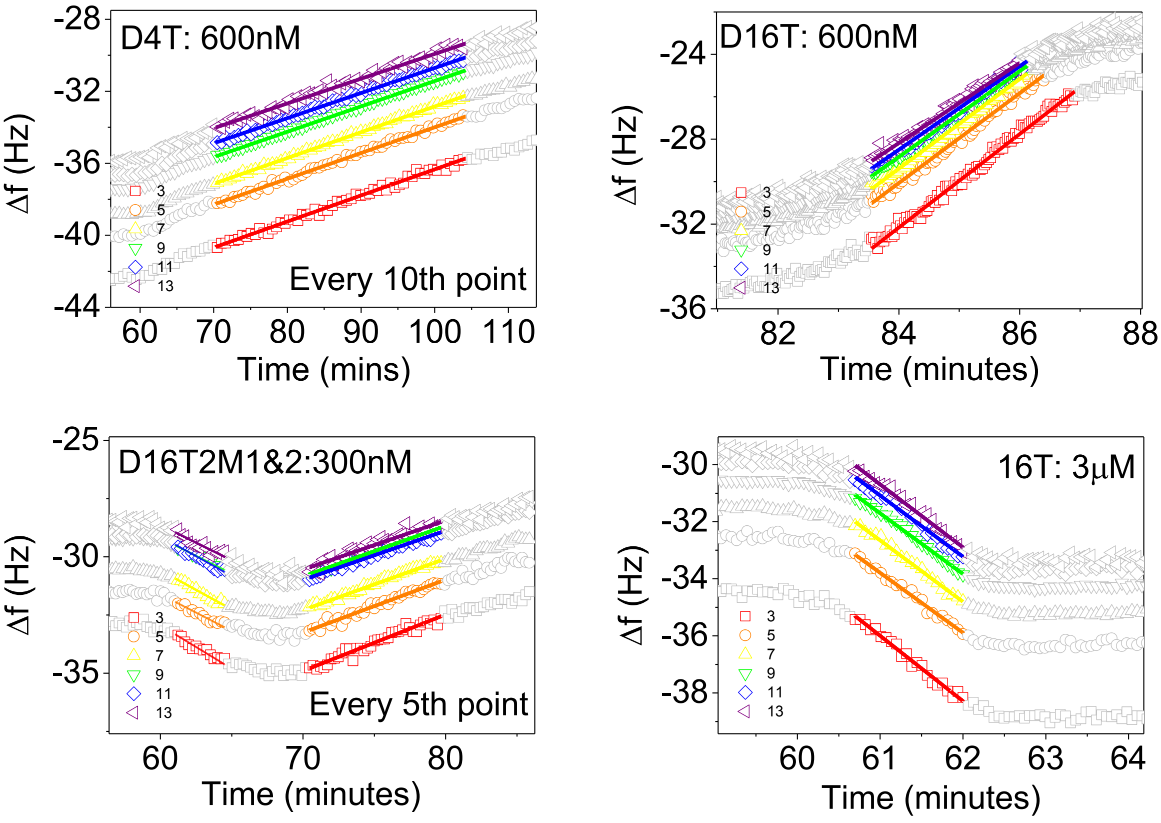

Fig. S3

Supplementary Table 3: Results of fits shown in Supplementary Figure 3

The table shows the gradient, the standard error and the Pearson correlation coefficient for the linear equations fitted to the data of Supplementary Figure 3.

D4T: 600nM			
	Gradient	Standard Error	Pearson
3	0.145	0.0005	0.997
5	0.143	0.0005	0.997
7	0.143	0.0003	0.999
9	0.141	0.0002	1.000
11	0.140	0.0003	0.999
13	0.138	0.0007	0.994
D16T: 600nM			
	Gradient	Standard Error	Pearson
3	2.204	0.0188	0.996
5	2.117	0.0233	0.995
7	2.113	0.0180	0.997
9	2.021	0.0133	0.998
11	1.968	0.0218	0.995
13	1.889	0.0296	0.991
D16T2M1,2: 300nM			
Phase 1			
	Gradient	Standard Error	Pearson
3	-0.372	0.0159	-0.954

5	-0.315	0.0133	-0.955
7	-0.334	0.0089	-0.981
9	-0.306	0.0062	-0.989
11	-0.341	0.0093	-0.981
13	-0.304	0.0178	-0.919
Phase 2			
	Gradient	Standard Error	Pearson
3	0.242	0.0036	0.984
5	0.228	0.0033	0.985
7	0.221	0.0021	0.993
9	0.217	0.0015	0.997
11	0.211	0.0023	0.991
13	0.211	0.0046	0.966
16T: 3μM			
	Gradient	Standard Error	Pearson
3	-2.290	0.0701	-0.991
5	-2.098	0.0525	-0.994
7	-2.151	0.0490	-0.995
9	-2.127	0.0379	-0.997
11	-2.141	0.0632	-0.991
13	-2.205	0.0740	-0.989

Supplementary Figure 4: histogram of residuals for fit to data for D16T (600nM)

The ith residual is defined as r_i=(*observed value of y*)$_i$-(*predicted value of y*)$_i$. The distribution of residual values is shown below for the case of a linear fit to the Δ*f(t)* trace for D16T at a concentration of 600nM. The residuals are normally distributed around a central value of 0, as indicated by the Gaussian fit shown as a solid black line.

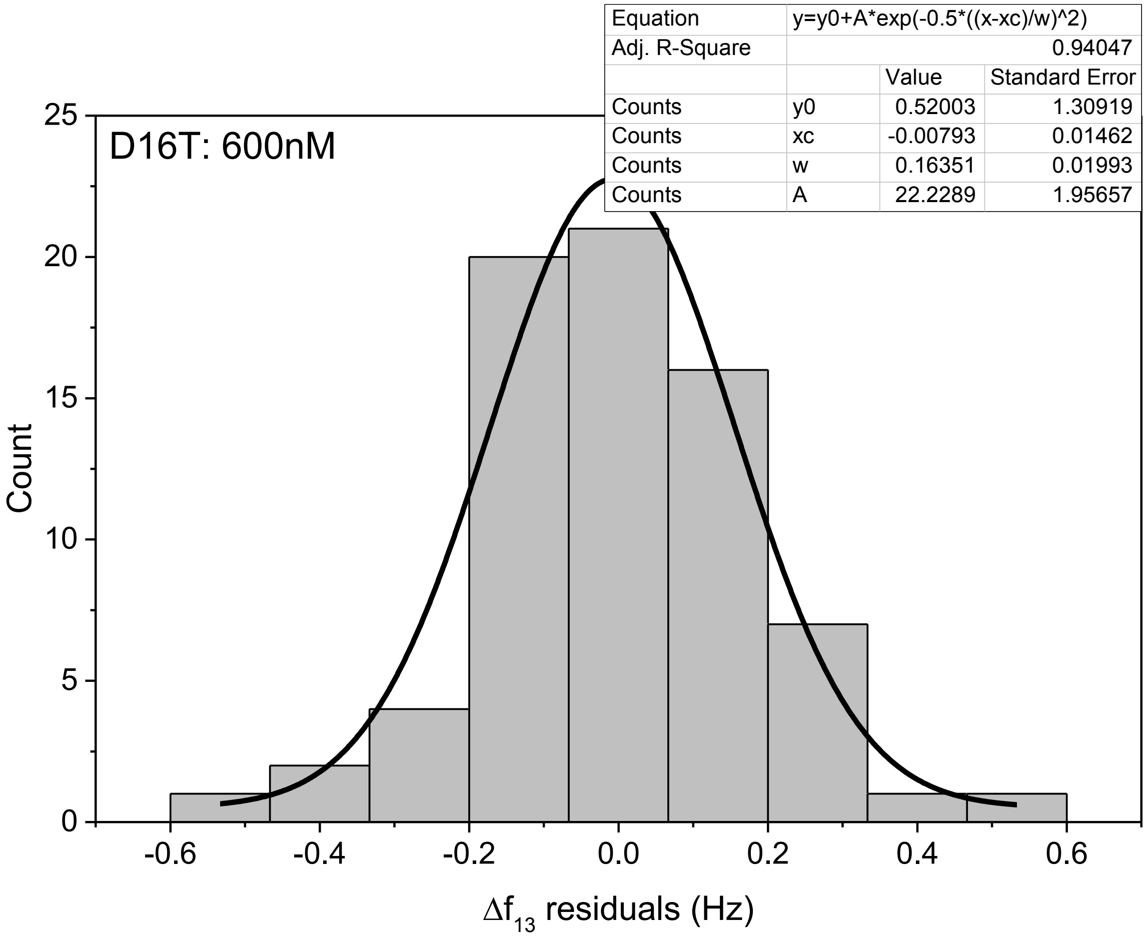

Fig. S4

Supplementary Figure 5: Residuals for fit (D16T, 600nM), as function of independent variable

The plot below shows the residuals from the previous figure as a function of the independent variable, time. The residuals are independent of time, indicating that the original data exhibited no systematic deviation from a linear fit. The residuals themselves have been fitted with a straight line, which is seen to have a gradient of zero (within the error).

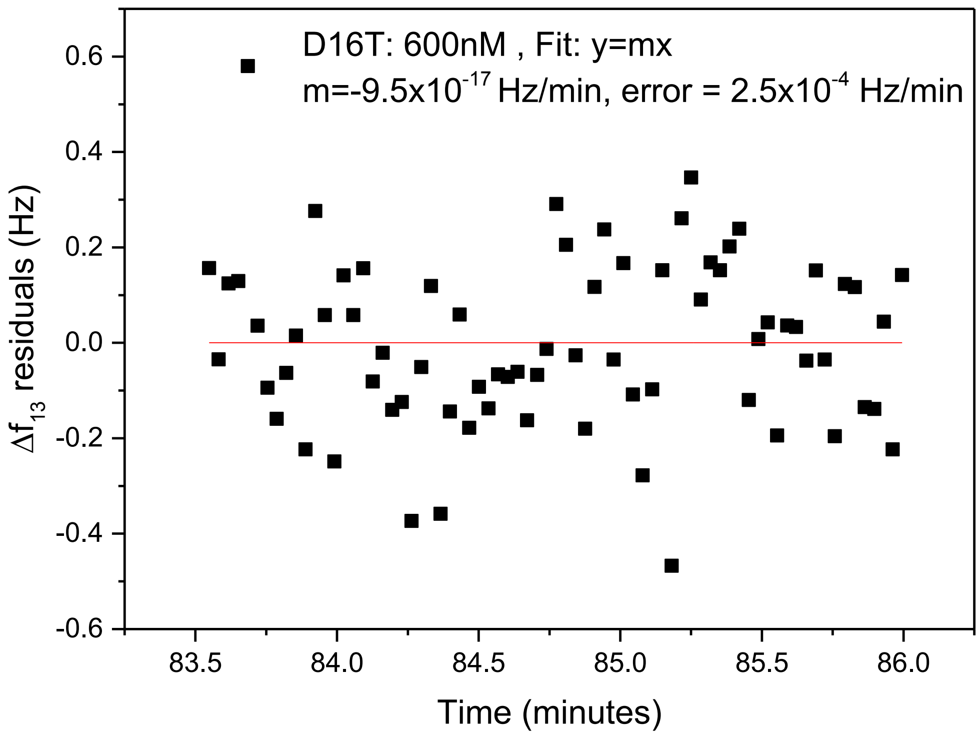

Fig. S5

Supplementary Figure 6: Effect of changing the density of molecules on the surface

Frequency shifts observed for experiments in which DNA machines were co-immobilized with MCH. Black points: data included in fit. Red line: result of fit using function $y=Ae^{-t/\tau}+B$. Each graph is labelled with the χ^2 values, the *p*-value for the Anderson-Darling test for normality of the fit residuals, and the fit parameter τ. The mixtures used for immobilization were prepared using stock solutions of DNA machines at 600nM and MCH at 1mM, mixed in various ratios. The DNA:MCH ratios selected were as follows: 1) 1:0; 2) 5:1; 3) 2:1; 4) 1:1; 5) 1:2; 6) 1:5. As the percentage of DNA is reduced, the quality of the exponential fit improves.

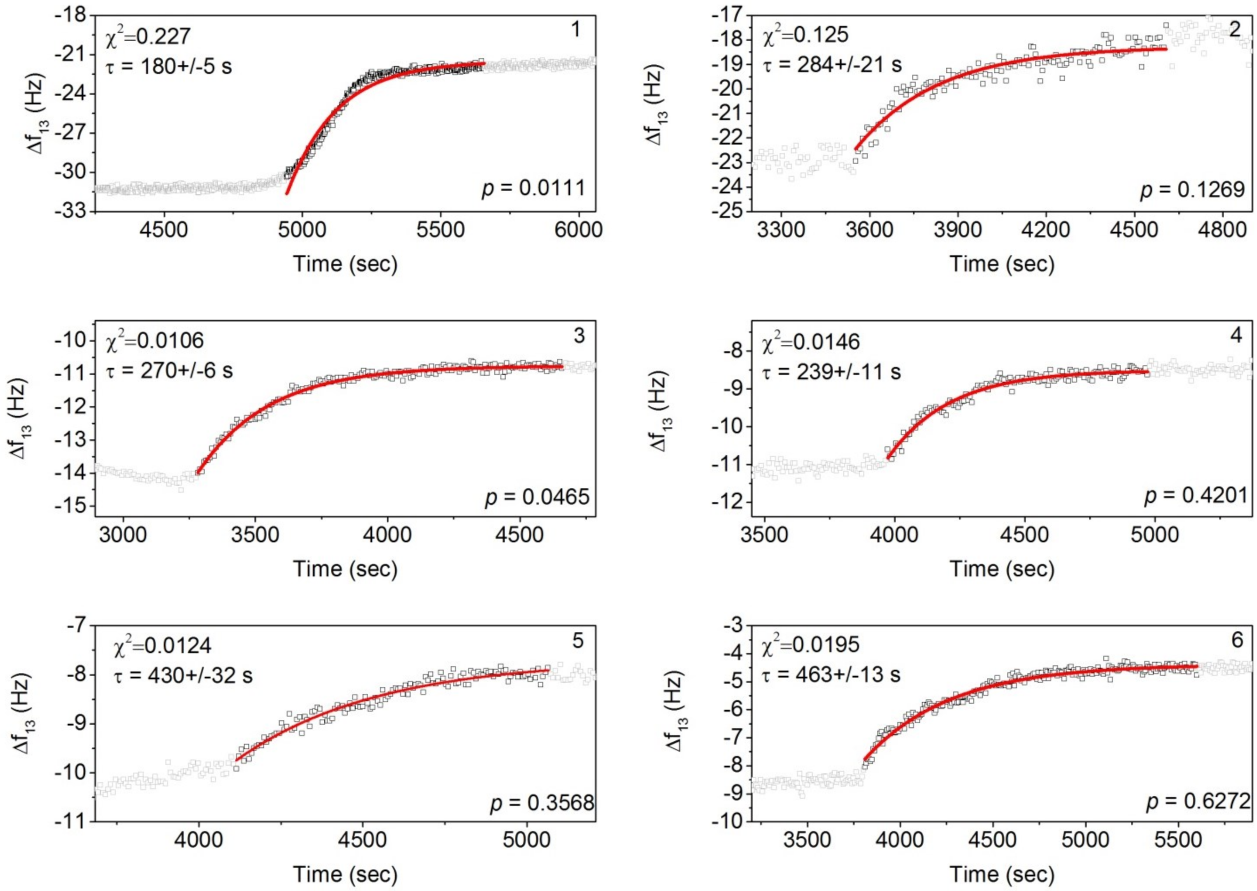

Fig. S6

Supplementary Figure 7: Strand displacement efficiency for invaders with no mismatches

(A) Efficiency of strand displacement as a function of invader concentration for D16T (16nt toehold and 16nt displacing domain – blue circles), 16T (16nt toehold, no displacing domain – purple diamonds) and D4T (4nt toehold and 16nt displacing domain – red squares). (B) Efficiency of strand displacement as a function of the length of the toehold binding domain, for an invader concentration of 600nM. In both graphs, the displacement efficiency is defined as frequency shift associated with binding or displacement divided by frequency shift due to immobilization. The thirteenth overtone was used and theoretical limits were estimated using the Sauerbrey equation. The frequency shift was obtained in each case by averaging the data over the plateau corresponding to the appropriate stage of the experiment and error bars correspond to the standard deviation of the averaged data. The edges of the plateau were identified manually.

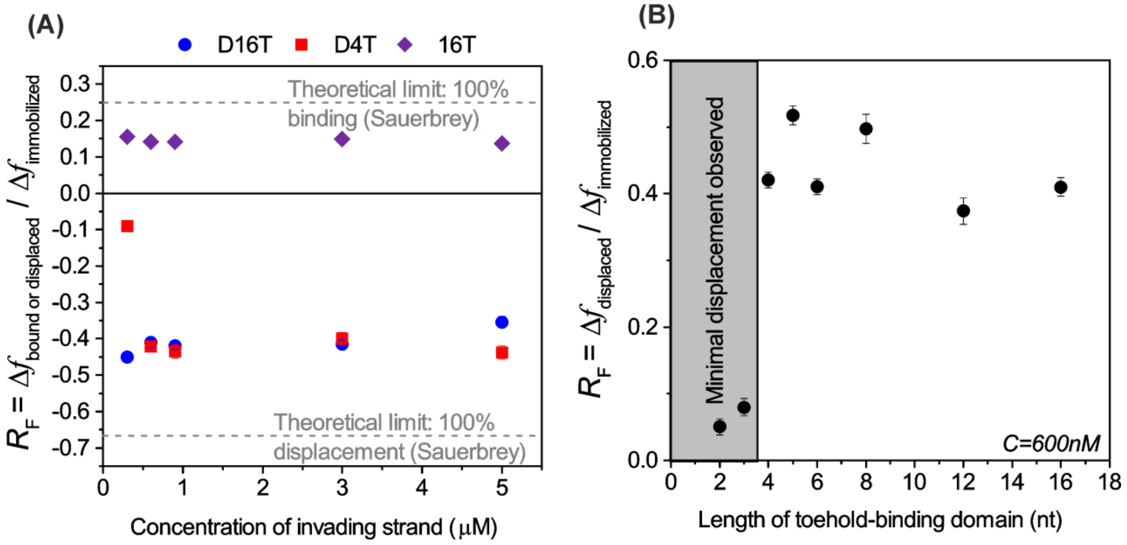

Fig. S7

Supplementary Figure 8: Minimal displacement observed when toehold binding domain is shorter than 4nt (with the original target sequence)

QCM-D measurements obtained for strand displacement experiments performed using invading strands with toehold binding domains 2 or 3 nucleotides in length.

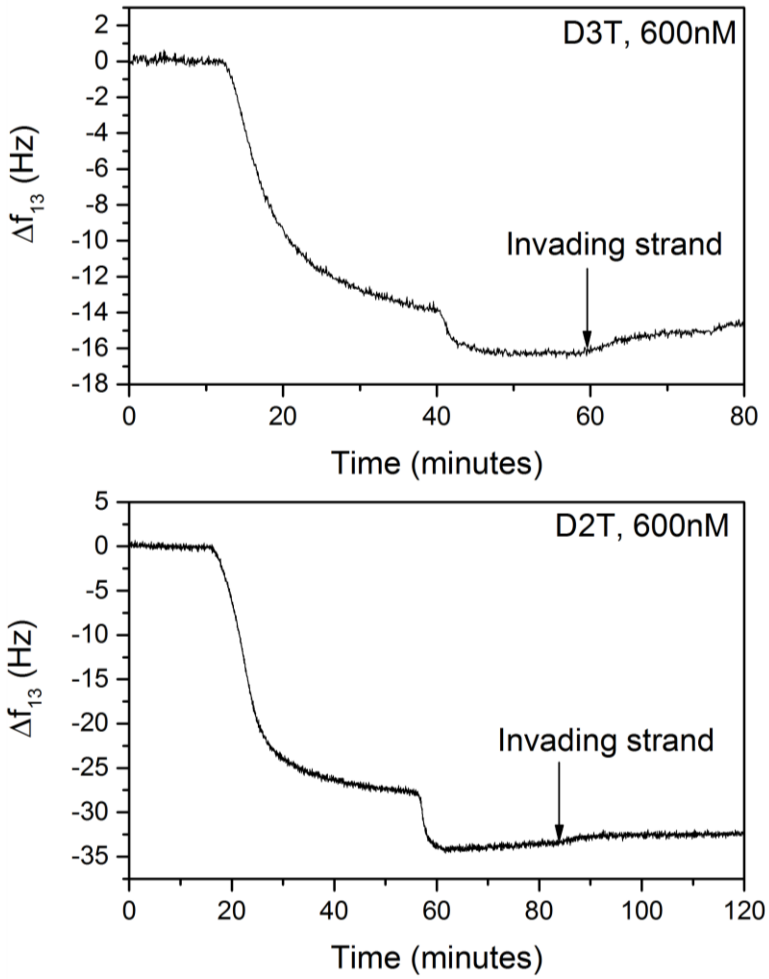

Fig. S8

Supplementary Figure 9: Strand displacement observed for invader with a 3nt toehold binding domain that is 100% C

QCM-D trace obtained for strand displacement experiment performed using invading strand with toehold binding domain 3 nucleotides in length, where the toehold is designed to be particularly strong and comprises 3 C bases.

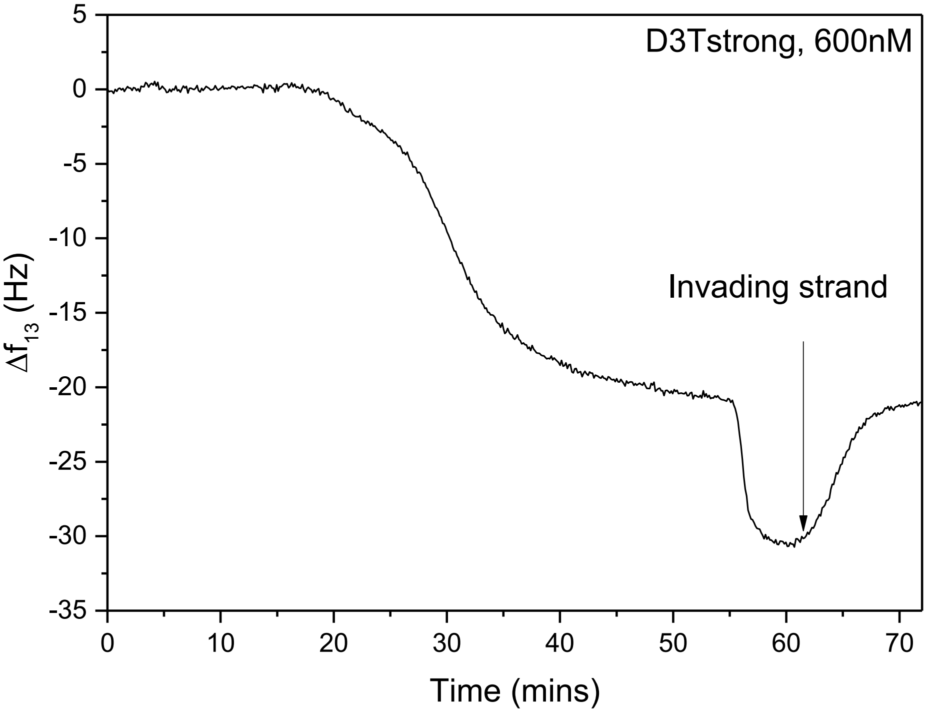

Fig. S9

Supplementary Discussion 3: Calculation of relative rates of strand displacement and toehold binding

The table below shows the rate of binding for 16T and the rate of displacement for D16T, measured for the 13th overtone, at a range of invader concentrations. 16T is 16nt long, while the target for D16T is 32nt long. Hence, when a single 16T invader binds to its target, the mass of the immobilized complex increases by 16nt. Similarly, when D16T initiates displacement of a target, the mass of the immobilized machine decreases by 32nt. Since the displacement rate is measured in units of Hz min^{-1}, it is necessary to normalize the ratio of the rates by dividing by the ratio of masses, giving rise to the values in the right-hand column. The mean value of the normalized ratio is 0.84.

**Concentration (μM)**	**v_{13}, 16T, binding (Hz min^{-1})**	**v_{13}, D16T, displacement (Hz min^{-1})**	**Ratio (D16T/16T)**	**Normalized ratio**
0.3	1.00	1.54	1.54	0.77
0.6	1.16	1.89	1.63	0.81
0.9	1.41	2.55	1.81	0.90
3	2.21	3.96	1.79	0.90
5	2.46	3.95	1.61	0.80

Supplementary Figure 10: Overtone-dependence of dissipation shift observed for binding of toehold-only invading strand

Measured changes in Δ*D* for experiment performed with toehold-only strand 16T, at a concentration of 5μM: traces are shown for all overtones as a function of time (illustrating immobilization of the nanomachines, backfilling and binding of the toehold-only strand), and the magnitude of the shift in ΔD observed upon binding is plotted explicitly. The shift corresponding to the thirteenth overtone is minimal.

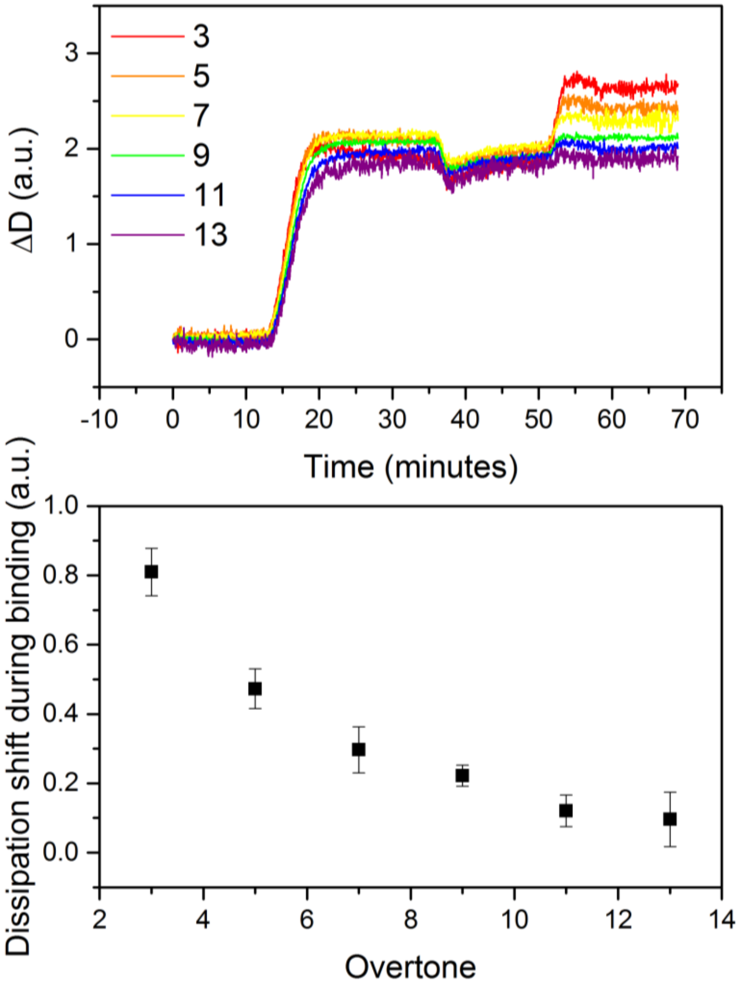

Fig. S10

Supplementary Figure 11: Control experiments

Top: Frequency change observed with QCM-D during consecutive application of input strands with no toehold binding domain and no complementarity with the target. The response is negligible, demonstrating that minimal blunt-end displacement occurs and that the presence of a specific input (the complementary strand) is necessary if strand displacement is to be initiated. Bottom: Frequency change observed with QCM-D for a strand with a toehold binding domain of 4 nucleotides in length and a mismatch in the first base of the displacing domain. Both figures: Concentration of invaders was 5μM.

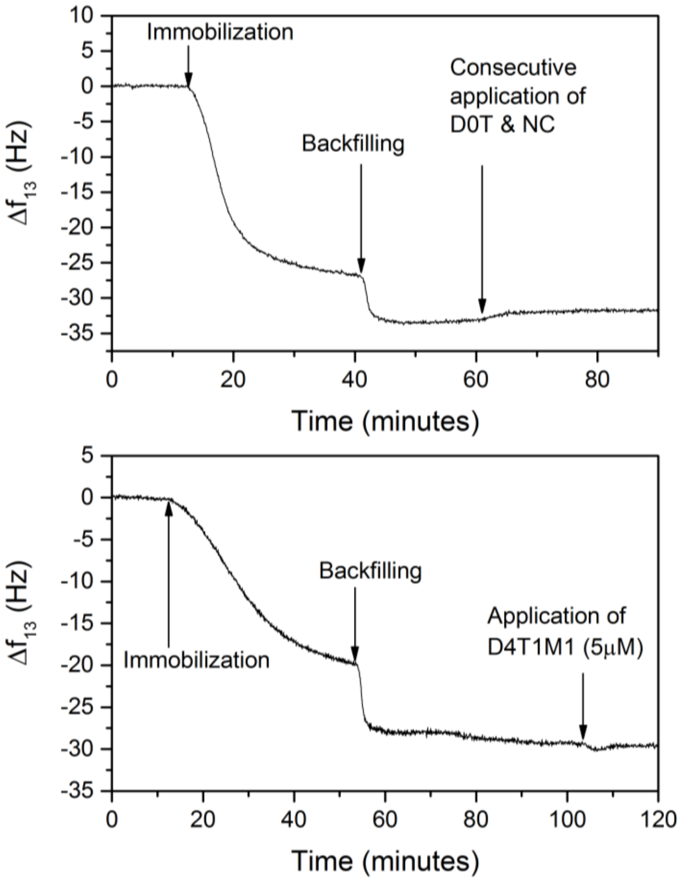

Fig. S11
